# Implicitly assessed attitudes toward body shape and food: the moderating roles of dietary restraint and disinhibition

**DOI:** 10.1186/s40337-015-0085-8

**Published:** 2015-12-08

**Authors:** Joanna Myriam Moussally, Joël Billieux, Olivia Mobbs, Stéphane Rothen, Martial Van der Linden

**Affiliations:** Psychology Department, FPSE, Cognitive Psychopathology and Neuropsychology Unit, University of Geneva, Boulevard du Pont d’Arve 40, CH-1205 Geneva, Switzerland; Swiss Center for Affective Sciences, Campus Biotech, University of Geneva, Case Postale 60, CH-1211 Geneva 20, Switzerland; Psychological Sciences Research Institute, Laboratory for Experimental Psychopathology, Catholic University of Louvain, Place Cardinal Mercier 10, B-1348 Louvain-la-Neuve, Belgium; Mental Health and Psychiatry Department, Addictology Division, Geneva University Hospitals, Rue Grand-Pré 70C, CH-1202 Geneva, Switzerland; Geneva School of Economics and Management, Research Center for Statistics, University of Geneva, Boulevard du Pont d’Arve 40, CH-1205 Geneva, Switzerland; Cognitive Sciences Department, Cognitive Psychopathology Unit, University of Liège, Boulevard du Rectorat B33 (TriFacultaire), 4000 Liège, Belgium

**Keywords:** Implicitly assessed attitudes, Food, Body shape, Restraint, Disinhibition

## Abstract

**Background:**

Attitudes toward body shape and food play a role in the development and maintenance of dysfunctional eating behaviors. Nevertheless, they are rarely investigated together. Therefore, this study aimed to explore the interrelationships between implicitly assessed attitudes toward body shape and food and to investigate the moderating effect on these associations of interindividual differences in problematic and nonproblematic eating behaviors (i.e., flexible versus rigid cognitive control dimension of restraint, disinhibition).

**Methods:**

One hundred and twenty-one young women from the community completed two adapted versions of the Affect Misattribution Procedure to implicitly assess attitudes toward body shape (i.e., thin and overweight bodies) and food (i.e., “permitted” and “forbidden” foods), as well as the Three-Factor Eating Questionnaire to evaluate restraint and disinhibition.

**Results:**

The results revealed that an implicit preference for thinness was positively associated with a positive attitude toward permitted (i.e., low-calorie) foods. This congruence between implicitly assessed attitudes toward body shape and food was significant at average and high levels of flexible control (i.e., functional component of eating). Moreover, an implicit preference for thinness was also positively associated with a positive attitude toward forbidden (i.e., high-calorie) foods. This discordance between implicitly assessed attitudes was significant at average and high levels of rigid control and disinhibition (i.e., dysfunctional components of eating).

**Conclusions:**

These findings shed new light on the influence of congruent or discordant implicitly assessed attitudes toward body shape and food on normal and problematic eating behaviors; clinical implications are discussed.

**Electronic supplementary material:**

The online version of this article (doi:10.1186/s40337-015-0085-8) contains supplementary material, which is available to authorized users.

## Background

Attitudes are representations stored in memory about the valence of a specific stimulus [1]. These valenced representations of body shape/weight and food seem to play an important role in the development and maintenance of dysfunctional eating behaviors [2, 3]. Indeed, in Western societies, thinness is valued and the thin-ideal body is promoted by the media, while excess weight is disapproved of [4]. Nevertheless, the thin-ideal body is difficult or impossible to achieve. Every woman has a minimum weight, defined by genetic and physiological influences (e.g., morphology); it is difficult to maintain a weight below that minimum and remain healthy [5]. Internalization of the thin-ideal, coupled with this divergence between cultural standards and biological constraints, promotes body dissatisfaction and problematic eating behaviors, such as inflexibly restrictive eating behaviors intended to lose weight [4]. In dietary restraint, foods are categorized as negative or positive, meaning that they are perceived as “forbidden” (usually high-calorie foods) or “permitted” (usually low-calorie foods) [6]. Thus, attitudes toward body shape/weight (i.e., valuing thinness) seem to influence attitudes toward foods (i.e., dichotomous categorization of foods), at least among women who are dissatisfied with their bodies and want to achieve their ideal thinness by dieting.

Attitudes have been extensively studied with two types of measurement – *explicit* and *implicit* – to better understand and predict behaviors. Explicit measures of attitudes (e.g., self-reports) tap into reflective processes activated with voluntary cognitive control, meaning that they depend on responses and behaviors that participants are aware of, willing to report, and able to modify. Conversely and complementarily, implicit measures of attitudes reflect automatic processes activated without awareness or the possibility of controlling or modifying them [1, 7]. It has been demonstrated that, in certain cases, implicitly and explicitly assessed attitudes predict different types of behaviors (spontaneous behaviors for implicitly assessed attitudes and intentional behaviors for explicitly assessed attitudes), but they can also interact to influence behaviors [8]. Indeed, implicitly assessed attitudes not only affect spontaneous behaviors but can also influence intentional behaviors [9], explaining a significant proportion of the variance in different behaviors (e.g., drinking) independently of explicitly assessed attitudes [10]. It has therefore been proposed that, under some conditions, implicitly assessed attitudes might predict behaviors better than explicitly assessed attitudes, due to the biases associated with explicit assessment [9, 10]. In the field of eating disorders, various researchers [11, 12] have promoted the examination of implicitly assessed cognitions to gain access to representations that participants feel uncomfortable about confessing (e.g., preference for emaciation) and to better understand how implicit representations (e.g., liking of high-calories foods) might influence the development and maintenance of problematic eating behaviors (e.g., overeating, rigid restraint).

Past studies of implicitly assessed attitudes toward body shape emphasized the existence of positive attitudes toward thinness and/or negative attitudes toward fatness in participants from the community [13, 14], restrained and unrestrained eaters [15], participants with a high drive for thinness [16], individuals with anorexia nervosa [17] and obese/overweight persons [18]. Studies that focused on implicitly assessed attitudes toward food produced more inconsistent results. Some studies observed positive attitudes toward low-calorie foods and/or negative attitudes toward high-calorie foods in participants from the community [19], obese persons [20], and restrained eaters [12]. Other studies, though, showed the reverse pattern: positive attitudes toward high-calorie foods and/or negative attitudes toward low-calorie foods. This effect has been found in normal-weight, overweight and obese persons [21], as well as in restrained and unrestrained eaters [22, 23].

A first explanation for these divergent results might be the influence of methodological factors, such as the type of task used. Almost all studies examining attitudes toward body shape employed a relative measure: the Implicit Association Task (IAT) [24]. In contrast, studies of attitudes toward food have used several tasks. Interestingly, studies that observed positive attitudes toward low-calorie foods and/or negative attitudes toward high-calorie foods used the IAT, while studies that obtained the reverse pattern of results used nonrelative measures. In relative measures, the strength of the association for a targeted concept (e.g., “thin and positive”) is connected to the strength of the association for another targeted concept (e.g., “overweight and negative”), meaning that a positive attitude toward thinness necessarily also reflects a negative attitude toward fatness [25]. Conversely, a nonrelative measure can reveal independent attitudes toward two targeted stimuli, allowing exploration of the presence of similar attitudes toward different types of stimuli (e.g., positive attitudes toward both permitted and forbidden foods), which might explain some of the inconsistent findings about attitudes toward food.

A second explanation might be the influence of interindividual differences. Stimulus valuation, for example, could be related to the type of problematic eating behaviors or cognitions a person has. Based on their literature review, Roefs et al. [3] hypothesized that individuals with bulimia nervosa, restrained eaters and obese persons would have positive implicitly assessed attitudes toward high-calorie foods, given that these individuals present episodes of disinhibited overeating. In contrast, individuals with restrictive anorexia nervosa (who do not exhibit disinhibited overeating) would present negative attitudes toward similar foods. Furthermore, the effects observed in overall samples might hide subgroup profiles. Ahern et al. [16] observed that, unlike the negative attitudes toward thinness found in their overall sample of young women from the community, a subgroup of participants with an elevated drive for thinness had positive attitudes. Accordingly, interindividual differences in the domain of eating disorders should be explored.

Although, as mentioned above, attitudes toward body shapes seem to influence attitudes toward foods, few studies have tested their interrelationships when these attitudes were implicitly investigated. However, associations between food and body image have been studied extensively with both explicit and indirect behavioral measures. Mere exposure to food stimuli (e.g., high-calorie foods) or food consumption (e.g., having a milkshake) increases the explicitly reported state dissatisfaction with body or weight, especially among participants who display food, weight and shape concerns, such as restrained eaters [26, 27]. Furthermore, exposure to body-shape-related stimuli may also impact explicitly reported feelings and motivational states toward food (e.g., increase in guilt about chocolate consumption after exposure to images of thin women) [28]. Based on these results, it seems essential to explore the relationships between body shape/weight and food in the field of implicitly assessed attitudes.

### Overview of the present study

This study had three objectives. We first wanted to reexamine, using a nonrelative measure, implicitly assessed attitudes toward body shape (i.e., thin versus overweight bodies) and food (i.e., “forbidden” high-calorie versus “permitted” low-calorie foods) among young women from the community in order to replicate previous consistent findings (studies about attitudes toward body shape) or to find possible explanations for inconsistent findings described in the literature (studies about attitudes toward food). Second and more importantly, we wanted to highlight relationships between attitudes toward body shape and food, given that both play an important role in dysfunctional eating behaviors [2, 3], but have rarely been investigated together. Finally, we wanted to explore the role of interindividual differences in eating behaviors as moderators of the relationships between attitudes to better understand implicit representation patterns (associations between body shape and food concepts) that might underline behaviors [12]. Specifically, we wished to examine variations in attitude association patterns (e.g., positive attitudes toward thinness and permitted foods; positive attitudes toward thinness and forbidden foods) depending on participants’ levels (high versus low) of problematic and nonproblematic behaviors.

Regarding this third objective, we focused on two behavioral and cognitive components of eating: restraint and disinhibition. Restraint refers to the tendency to voluntarily restrict food intake by cognitively controlling eating behavior [29]. Westenhoefer [29] proposed dividing restraint into two manifestations: (a) flexible cognitive control (a nuanced attitude toward eating, where “forbidden” foods can be eaten in small quantities) is a more functional component, while (b) rigid cognitive control (a dichotomous, all-or-nothing attitude toward eating) is a more dysfunctional component. Disinhibition reflects the tendency to lose control over eating behaviors while experiencing adverse emotional states [29]. Consideration of restraint and disinhibition is relevant, as they are widely described as “characteristic features” of people with eating disorders and are transdiagnostic (found across different eating disorders) [30]. These components were described among restrained eaters and patients with bulimia or anorexia nervosa who tend to have episodes of disinhibited overeating between their restraint periods [30, 31]. Furthermore, these components are interconnected: rigid cognitive control was associated with high levels of disinhibition [29]. Finally, considering the two manifestations of restraint allows us to focus on the different mechanisms that underlie dieting behavior. Indeed, Westenhoefer [29] highlighted that the homogeneity of the restraint construct is questionable. He pointed out that all restrained eaters do not present episodes of disinhibited overeating; some succeed in restraint (probably because they have established more adaptive strategies). Therefore, assessing flexible and rigid cognitive controls might allow us to dissociate attitude patterns that underline the two different types of restraint.

## Methods

### Participants

One hundred and twenty-one young women took part in the study. All participants were volunteers and were recruited from the community in Geneva, Switzerland (41.32 % of the sample), and Liège, Belgium (58.68 %). Their ages ranged from 19 to 37 years old (*M* = 23.97, *SD =* 4.80) and their body mass indexes (BMI) from 15.73 to 35.86 (*M* = 21.38, *SD* = 3.40). All participants gave their consent prior to starting the experiment. The study protocol was approved by the Ethical Committee of the Psychology Department of the University of Geneva and carried out according to the 1964 Declaration of Helsinki.

### Measures

#### Personal information

Various questions were asked about participants’ sociodemographic and anthropometric data (e.g., age, height, weight). The self-reported height and weight were used to calculate the participants’ BMI.

#### Implicitly assessed attitudes

Attitudes toward body shape and food were assessed with two versions of the Affect Misattribution Procedure (AMP), adapted from Payne et al. [32]. The AMP was chosen because it is a nonrelative measure. It thus allowed us to explore the presence of independent attitudes toward two targeted stimuli (thin versus overweight bodies; permitted versus forbidden foods). It has also increasingly been used in various research fields (e.g., drinking or eating behaviors) [10, 33] and is characterized by elevated internal consistency (Cronbach’s alphas: .73 to .95). The construct validity of the AMP is supported by previous studies showing dissociations between implicit attitudes toward universally positive versus negative stimuli and associations with explicit attitude measures [10, 32]. Moreover, AMP-assessed attitudes predict judgments and behavioral outcomes even when explicit measures are controlled for, highlighting that AMP-assessed attitudes explain unique variance independently of explicit measures, which supports satisfactory predictive validity [10].

In AMP trials, an image (e.g., an emotional prime) appeared briefly (75 ms) in the center of the screen. Immediately following its disappearance, a blank screen appeared for 125 ms, and then an ambiguous pictograph (a Chinese character that is not familiar and therefore meaningless to participants)^1^ for 100 ms. After the pictograph disappeared, a pattern mask appeared and remained on the screen until the participant indicated the pleasantness (valence judgment) of the Chinese character by pressing a key. Participants were instructed to press a key labeled “pleasant” if they evaluated the character to be more pleasant than average and a key labeled “unpleasant” if they evaluated it to be less pleasant than average. The outcome variables were the proportion of Chinese characters evaluated as pleasant in each prime condition.

The AMP is based on people’s tendency, in ambiguous conditions, to erroneously attribute their feelings to one source (i.e., the pictograph) when they actually emanate from another one that is close in space and time (i.e., the emotional prime) [32]. Accordingly, judgment of the pictograph is influenced by the emotional prime preceding it. The participants were thus instructed that the picture preceding the Chinese character might influence their judgment of the pictograph and told that they should do their best not to let that happen.

The two created AMP versions were similar except for the stimuli (i.e., primes) used. They were composed of 72 randomly ordered trials consisting of three different types of primes; 72 different Chinese characters were used as targets in each task.

In the “body shape” version, the three types of primes consisted of (a) full-body pictures of thin women (*thin* primes; approximate BMIs located within the “moderate thinness” and “normal range” classes corresponding to 16.00–24.99 kg/m^2^), (b) full-body pictures of overweight women (*overweight* primes; approximate BMIs located within the “overweight” and “moderate obesity” classes corresponding to 25.00–39.99 kg/m^2^), and (c) pictures of shrubs (*control* primes). The pictures of thin and overweight women did not differ in clothing or skin color. Each prime condition contained 12 different pictures, which were presented twice. The images were evaluated, with 9-point Likert scales, in a pilot study of 40 women aged 20 to 35 years old. The results revealed that the pictures of thin and overweight women differed in fatness (median diff. = 3.80; Mann–Whitney *U*-test; *z* = 4.13, *p* < .001),^2^ but not in beauty (mean diff. = 0.25, 95 % CI = [−.40, .89], *t*(22) = .80, *p* = .434, *d* = .325).

In the “food” version, the three types of primes consisted of (a) pictures of “low-calorie” foods (*permitted* primes; e.g., vegetables), (b) pictures of “high-calorie” foods (*forbidden* primes; e.g., hamburger), and (c) pictures of everyday objects (*control* primes; e.g., bucket). Once again, each prime condition contained 12 different pictures. The pilot study mentioned above showed that permitted and forbidden foods differed significantly in the “forbidden” value they conveyed (median diff. = 6.90; Mann–Whitney *U*-test; *z* = 4.13, *p* < .001).^2^

The decision to use control stimuli other than the neutral gray square used by Payne et al. [32] was made to avoid repetition of the same stimulus throughout the task, which might lead to a habituation effect. In our study, each control stimulus appeared the same number of times as an emotional stimulus (*thin, overweight, permitted* and *forbidden* primes). Moreover, the use of images for the control stimuli increased the task’s ecological validity.

#### Eating behaviors

Restraint and disinhibition were measured with the French version [34] of the Three-Factor Eating Questionnaire (TFEQ) [35]. Restraint is measured with 21 items scored 0 (false) or 1 (true) and disinhibition is assessed with 16 items.^3^ The internal reliability of the two dimensions in the present sample was satisfactory (Cronbach’s alphas were respectively .88 and .74). As the objective of this study was to examine the roles of the different restraint manifestations, we calculated the scores for the rigid (7 items) and flexible (7 items) cognitive control dimensions proposed by Westenhoefer [29]. The internal reliability of the two subdimensions was acceptable (Cronbach’s alphas were respectively .76 and .65).

### Procedure

After giving their informed consent, participants completed the personal information sheet. Then they were randomly assigned to an order for the AMP versions: half of the participants started with the body shape version and the other half with the food version. Afterward, participants completed the TFEQ and were subsequently debriefed.

### Hypotheses and statistical analyses

#### Attitudes in the overall sample

Our first objective was to examine attitudes in the overall sample (i.e., mean level of AMP performances). Concerning attitudes toward body shape, we expected to replicate the findings obtained in community samples (i.e., positive attitudes toward thinness and negative toward fatness) [13]. For attitudes toward food, given that previous findings from community samples are inconsistent [19, 21], we had no a priori hypothesis. Indeed, the inconsistent findings described in the literature might suggest either dissimilar attitudes (positive attitudes toward permitted foods and negative toward forbidden foods) or similar attitudes (positive attitudes toward permitted and forbidden foods).

To meet this objective, we computed repeated measures analyses of variance (ANOVAs) on the AMP performances (proportion of pleasant judgments in each prime condition) separately for the “body shape” and “food” versions. We used Tukey’s post hoc tests to test the differences in evaluative responses between the conditions. All mean levels of AMP performances were then compared with the chance level of .50 to reinforce findings from prior Tukey’s post hoc tests. Indeed, in determining the valence of an attitude, two conditions are required. First, the proportion of pleasant judgements in the emotional conditions has to significantly differ from the control condition (highlighted by Tukey’s post hoc tests). Second, the proportion of pleasant judgements has to differ from .50. Conversely, it is expected that the pleasant judgements in the control condition are about .50 [10, 32]. For these analyses, the conventional significance level was adjusted for multiple comparisons with the Bonferroni correction.

#### Relationships between attitudes

Concerning our second objective of highlighting relationships between attitudes toward body shape and food, we were particularly interested in the valuing of thinness (compared with fatness), given the antagonist valences assigned to thinness and excess weight in Western societies and the central role of thin-ideal internalization (preference for thinness) in food categorization and eating behaviors [4, 6]. We therefore posited that attitudes toward thinness would be positively related to attitudes toward “permitted” foods and negatively related to attitudes toward “forbidden” foods. Indeed, in order to lose weight, an individual will categorize foods as “positive/permitted” and “negative/forbidden” [6].

Interrelationships among attitudes were explored with Pearson correlations. For these analyses, we computed AMP scores corresponding to the guidelines provided by Payne et al. [10, 32, 36]: the AMP can be scored either as a relative preference score (relative preference for a type of stimuli over another) or as an independent attitude score (positivity toward a type of stimuli).

In the “body shape” version, we were interested in the relative preference for thin or overweight bodies (*AMP-assessed attitude toward body shape*), given that we particularly wanted to examine the valuing of thinness. Therefore, the proportion of pleasant responses on *overweight* trials was subtracted from the proportion of pleasant responses on *thin* trials. A positive score indicates a preference for thin bodies.

For the “food” version, we created three different scores because, as mentioned above, unlike studies in the domain of implicitly assessed attitudes toward body shape, which present quite consistent findings, studies in the field of attitudes toward food have produced more inconsistent results. Therefore, we computed (a) a score of relative preference for permitted or forbidden foods (*AMP-assessed attitude toward food*), and (b) two independent scores of attitudes (i.e., *AMP-assessed attitude toward permitted foods* and *AMP-assessed attitude toward forbidden foods*) since we expected that different types of foods might receive the same implicit judgment. For the AMP-assessed attitude toward food, responses on *forbidden* trials were subtracted from responses on *permitted* trials. A positive score indicates a relative preference for permitted foods. For the AMP-assessed attitude toward permitted foods, responses on *control* trials were subtracted from responses on *permitted* trials. A positive score indicates a preference for permitted foods. Finally, for the AMP-assessed attitude toward forbidden foods, responses on *control* trials were subtracted from responses on *forbidden* trials. The consideration of *control* trials in the creation of independent attitude scores allows interindividual differences in responses to be taken into account (e.g., tendency to respond in a similar way through prime conditions, such as showing “pleasant responses”).

Pearson correlations were run between the relative preference score obtained for the “body shape” version and the three scores obtained for the “food” version. To take multiple testing into account, we used the Bonferroni correction. Moreover, as responses on *control* trials in the “body shape” version were not included in any of the AMP scores, their effect was controlled for (by being holding constant) in order to take into account interindividual differences in responses. Therefore, in the Results section, we report partial correlations.

#### Interindividual differences in eating behaviors

Regarding this last objective of exploring the role of eating behaviors as moderators of the relationships between attitudes toward body shape and food, two divergent assumptions might be proposed. First, in line with the hypothesized interrelationships postulated above (positive association between attitudes toward thinness and permitted foods; negative association with forbidden foods), the strength of associations between attitudes might be higher at high levels of dysfunctional components of eating (rigid restraint and disinhibition) than at low levels. Nevertheless, our hypothesized interrelationships describe congruent attitudes. Therefore, it is also conceivable that, at high levels of dysfunctional components, the interrelationships would illustrate discordance between attitude patterns. More specifically, Roefs et al. [3] proposed that people who alternated between restraint and disinhibition would present positive implicitly assessed attitudes toward high-calorie foods. Because these persons probably also display positive implicitly assessed attitudes toward thinness [12], their attitudes can be considered to be discordant.

For this objective, several multiple linear regression models were performed. The dependent variable was one of the scores obtained on the “food” version (see Results section for details). This choice was based on the assumption that attitudes toward foods might be influenced by attitudes toward body shapes. Therefore, the independent variables were (a) AMP-assessed attitude toward body shape; (b) the potential moderating variable (flexible or rigid control dimension of restraint, or disinhibition);^4^ and (c) the interaction term.^5^ Once again, performance on *control* trials in the “body shape” version was taken into account by entering this variable in the regression models as a control variable. When an interaction term was significant, it was further analyzed by using the method proposed by Aiken and West [37] in order to check whether high versus low levels of problematic or nonproblematic behaviors influence attitude associations (see Results section for details). The normality, linearity and homoscedasticity assumptions of residuals were satisfied for all regression models and no multicollinearity problems were detected (smallest tolerance value = .82).

## Results

### Attitudes in the overall sample

#### Body shape AMP

A significant main effect of prime type was observed (*F*(1.92, 230.75) = 4.37, *p* = .015, η^2^ = .035). Post hoc tests showed that participants judged the pictographs to be significantly (*p* = .011) more “pleasant” on *control* trials (*M* = 0.54, *SD* = 0.19) than on *overweight* trials (*M* = 0.48, *SD* = 0.18). Responses on *thin* trials (*M* = 0.50, *SD* = 0.18) did not differ significantly from either responses on *control* trials or responses on *overweight* trials (both *p*s > .05). Mean levels of AMP performance were then compared with the chance level of .50 (corrected alpha error level resulting from the Bonferroni correction: *p* = .017). Neither responses on *thin* trials nor responses on *overweight* trials nor responses on *control* trials differed significantly from the chance level (all *p*s > .017). Two differences are necessary to reach a conclusion about the valence of attitudes (i.e., positive or negative): a significant difference between emotional and control stimuli, and a difference from chance level. Thus, overweight and thin bodies seemed to be implicitly considered as ambiguous (i.e., without a defined valence) by our sample, even though the overweight bodies showed an interstimulus tendency to be judged as more negative than control primes.

#### Food AMP

A significant main effect of prime type was obtained (*F*(1.92, 230.08) = 3.23, *p* = .044, η^2^ = .026).^6^ Post hoc tests showed that participants judged the pictographs to be significantly (*p* = .034) more “pleasant” on *permitted* trials (*M* = 0.57, *SD* = 0.18) than on *control* trials (*M* = 0.53, *SD* = 0.18). Responses on *forbidden* trials (*M* = 0.56, *SD* = 0.21) did not differ significantly from either responses on *permitted* trials or responses on *control* trials (both *p*s > .05). Mean performance levels were then compared with the chance level of .50 (corrected alpha error level for multiple comparisons: *p* = .017). Responses on *permitted* trials (*t*(120) = 4.10, *p* < .001) and responses on *forbidden* trials (*t*(120) = 2.94, *p* = .004) differed significantly from the chance level, but responses on *control* trials did not (*t*(120) = 1.65, *p* = .102). Therefore, the sample’s attitudes toward permitted foods could be considered as positive. Attitudes toward forbidden foods must be considered as ambiguous, given that they did not differ from responses in the control condition; still, they showed a tendency to be positive as they differed from the chance level.

### Relationships between attitudes

Pearson correlations between the different AMP scores – after partialing out the effect of responses on *control* trials in the “body shape” AMP – are shown in Table 1. Interestingly, the AMP-assessed attitude toward body shape was not significantly correlated with the AMP-assessed attitude toward food. However, it was positively correlated with the AMP-assessed attitude toward permitted foods and the AMP-assessed attitude toward forbidden foods. Finally, these two independent scores were also positively correlated.

**Table 1:** Partial correlations between attitudes toward body shape and food

	1	2	3	4
1. AMP: “body shape”	–			
2. AMP: “food”	–.119	–		
3. AMP: “permitted foods”	.287*	.421*	–	
4. AMP: “forbidden foods”	.361*	–.643*	.424*	–

*Note. N* = 121. * *p* ≤ .001. The Bonferroni correction was used in these analyses, resulting in a corrected alpha error level of *p* = .008. The partial variable is the proportion of “pleasant” responses on *control* trials in the “body shape” AMP. AMP: “body shape” = relative preference for thin or overweight bodies; AMP: “food” = relative preference for permitted or forbidden foods; AMP: “permitted foods” = preference for permitted foods over control images in the “food” AMP; AMP: “forbidden foods” = preference for forbidden foods over control images in the “food” AMP

These results mean that the stronger participants’ relative implicit preference was for *thin* bodies (over *overweight* bodies), the stronger their positive implicitly assessed attitudes toward both *permitted* and *forbidden* foods also were. Moreover, the more positive the participants’ attitude toward *permitted* foods was, the more positive their attitude also was toward *forbidden* foods. Taken together with performance in the “food version” of the AMP (i.e., positive attitudes toward permitted foods and a tendency to make pleasant responses on forbidden trials), this finding shows that the different types of foods seemed to elicit similar (i.e., positive) attitudes in the overall sample. This could explain why the correlation between the two relative preference scores was not significant, and suggests that the use of a relative preference score toward food is not appropriate. This AMP score (*AMP-assessed attitude toward food*) will therefore not be included in further analyses.

### Interindividual differences in eating behaviors

To examine interindividual differences in eating behaviors, we thus focused on the relationships between the AMP-assessed attitude toward body shape and the two independent AMP-assessed attitudes toward foods. The dependent variable of the regression models was therefore the AMP-assessed attitude toward either permitted foods or forbidden foods. The results of the regression models for the mean levels of eating behaviors are displayed in Table 2.

**Table 2:** Linear regressions between attitudes toward body shape and food at mean level of eating behaviors

	Permitted foods				Forbidden foods			
*Regression models 1 and 2*	b* ^a^	*SE* ^b^	*t*(116)	*p*	b* ^a^	*SE* ^b^	*t*(116)	*p*
AMP-assessed attitude toward body shape	.296	.090	3.29	< .01	.370	.087	4.24	< .001
Flexible cognitive control	–.175	.088	−1.98	< .05	–.107	.086	−1.25	ns
Interaction term	.178	.087	2.03	< .05	.147	.085	1.74	ns
*Regression models 3 and 4*	b* ^a^	*SE* ^b^	*t*(115)^c^	*p*	b* ^a^	*SE* ^b^	*t*(116)	*p*
AMP-assessed attitude toward body shape	.192	.091	2.10	< .05	.318	.084	3.77	< .001
Rigid cognitive control	–.135	.094	−1.43	ns	–.086	.085	−1.00	ns
Interaction term	–.035	.091	–.38	ns	.230	.083	2.76	< .01
*Regression models 5 and 6*	b* ^a^	*SE* ^b^	*t*(115)^c^	*p*	b* ^a^	*SE* ^b^	*t*(116)	*p*
AMP-assessed attitude toward body shape	.195	.093	2.09	< .05	.251	.086	2.94	< .01
Disinhibition	.088	.096	.92	ns	.035	.086	.41	ns
Interaction term	.067	.097	.70	ns	.352	.087	4.03	< .001

*Note.* N = 121. In the six multiple regression models, the proportion of “pleasant” responses on *control* trials in the “body shape” AMP was entered as a control variable. The moderating variable was respectively (1) flexible cognitive control dimension of restraint, (2) rigid cognitive control dimension of restraint, and (3) disinhibition, resulting in the creation of three interaction terms: (1) “AMP-assessed attitude toward body shape × flexible control”, (2) “AMP-assessed attitude toward body shape × rigid control”, and (3) “AMP-assessed attitude toward body shape × disinhibition”. ^a^Standardized regression coefficients. ^b^Standard errors of b*. ^c^Degrees of freedom = 115, because of the exclusion of a participant with a Cook’s distance > 1.00

#### Flexible cognitive control dimension of restraint

At the mean level of flexible control, the AMP-assessed attitude toward body shape, the flexible control dimension and the interaction term “AMP body shape × flexible control” were significant predictors of the AMP-assessed attitude toward permitted foods (see Table 2). The overall model fit had a medium effect size (*R*^*2*^*=* .154, 95 % CI = [.041, .267]). To further analyze this significant two-way interaction and determine when the AMP-assessed attitude toward body shape is a significant predictor of the AMP-assessed attitude toward permitted foods, we checked whether the main effect of the AMP-assessed attitude toward body shape was significant at high and low levels (i.e., ±1 *SD*) of the moderator [37].^7^ The AMP-assessed attitude toward body shape was a significant predictor of the AMP-assessed attitude toward permitted foods at a high level of flexible control (b* = .469, *SE* = .136, *t*(116) = 3.45, *p* = .001),^8^ but not at a low level (b* = .122, *SE* = .111, *t*(116) = 1.10, *p =* .274). Therefore, when flexible control was average or high, the more the participants presented a relative implicit preference for *thin* bodies, the more positive their attitude was toward *permitted* foods. The intensity of the relationship between attitudes was stronger when flexible control was high than when it was average (i.e., increased *standardized beta coefficient*). The two-way interaction is illustrated in Additional file 1. The AMP-assessed attitude toward body shape was also a significant predictor of the AMP-assessed attitude toward forbidden foods; nonetheless, the interaction term “AMP body shape × flexible control” was not (see Table 2).

#### Rigid cognitive control dimension of restraint

At the mean level of rigid control, the AMP-assessed attitude toward body shape was a significant predictor of the AMP-assessed attitude toward permitted foods, but the interaction term “AMP body shape × rigid control” was not. However, both were significant predictors of the AMP-assessed attitude toward forbidden foods (see Table 2). The AMP-assessed attitude toward body shape was significant at a high level of rigid control (b* = .559, *SE =* .111, *t*(116) = 5.05, *p* < .001), but not at a low level (b* = .076, *SE =* .131, *t*(116) = .58, *p =* .563). Thus, when rigid control was average or high, the more the participants presented a relative implicit preference for *thin* bodies, the more positive their attitude was toward *forbidden* foods. The intensity of the relationship between attitudes was stronger when rigid control was high. The overall model fit had a medium effect size (*R*^*2*^*=* .228, 95 % CI = [.103, .354]). The two-way interaction is illustrated in Additional file 1.

#### Disinhibition

At the mean level of disinhibition, the AMP-assessed attitude toward body shape was a significant predictor of the AMP-assessed attitude toward permitted foods, but the interaction term “AMP body shape × disinhibition” was not. However, once again, both were significant predictors of the AMP-assessed attitude toward forbidden foods (see Table 2). The AMP-assessed attitude toward body shape was significant at a high level of disinhibition (b* = .504, *SE* = .091, *t*(116) = 5.54, *p* < .001), but not at a low level (b* = −.001, *SE* = .119, *t*(116) = −.01, *p =* .991). Therefore, when disinhibition was average or high, the more the participants presented a relative implicit preference for *thin* bodies, the more positive their attitude was toward *forbidden* foods. The intensity of the relationship between attitudes was stronger when disinhibition was high. The overall model fit had a large effect size (*R*^*2*^*=* .273, 95 % CI = [.144, .403]). The two-way interaction is illustrated in Additional file 1.

## Discussion

This study reexamined implicitly assessed attitudes toward body shape and food in a sample of young women from the community; more importantly, it also investigated the interrelationships between attitudes toward body shape and food as well as the moderating role of the flexible and rigid cognitive control dimensions of restraint, and disinhibition. Given the lack of studies investigating implicitly assessed attitudes toward body shape and food together, the main contribution of the present study is the examination of congruence and discordance in patterns of implicitly assessed attitudes in the understanding of problematic and nonproblematic eating behaviors.

The current study did not replicate earlier findings of positive attitudes toward thinness and negative attitudes toward fatness [13]. We found that both overweight and thin bodies were perceived as implicitly ambiguous (without a defined valence). As for attitudes toward food, a positive implicitly assessed attitude toward permitted foods was detected, replicating some previous findings [19]. Attitudes toward forbidden foods did not present a defined valence. However, these results are not surprising. First, it is conceivable that interindividual differences masked effects in the overall sample. Indeed, AMP performances in our sample presented considerable variability. The undefined valences obtained for attitudes toward forbidden foods and both body types could therefore be explained by the presence of incompatibly valenced attitudes (i.e., positive and negative) toward a single type of stimuli among participants, which canceled out the overall effects. The consideration of interindividual differences in the present sample highlighted some specific attitude patterns in subgroups of participants, as past studies had also found. For example, Ahern et al. [16] reported that participants could be characterized by either negative or positive attitudes toward thin bodies depending on their level of drive for thinness. Second, as mentioned, past studies obtained inconsistent results concerning forbidden foods [20, 23]; the same was also true of body shape. Roddy et al. [14], for instance, also failed to detect anti-overweight attitudes using a nonrelative measure like the AMP, underlying the influence of the task. Finally, in our overall sample, the different types of food seemed to elicit similar (positive) implicitly assessed attitudes rather than dissimilar attitudes. This result could account for some previous inconsistent findings. Indeed, the use of the IAT (relative measure) in several studies made it impossible to identify a similar pattern of attitudes for two targeted stimuli.

Regarding interrelationships between attitudes, the positive correlation observed between attitudes toward thinness and permitted foods supports our hypothesis. However, we did not expect that attitudes toward thinness would be positively – and not negatively – correlated with attitudes toward forbidden foods. This discordance between attitudes (i.e., positive association, whereas forbidden foods should be devalued to promote thinness) became meaningful when interindividual differences were considered. Indeed, their exploration revealed different patterns of attitudes toward body shape and food at average and high levels of problematic versus nonproblematic eating behaviors. More specifically, the functional component of restraint (i.e., flexible cognitive control) contributes to a positive relationship between attitudes toward thinness and permitted foods (*congruent* attitudes), whereas dysfunctional eating behaviors (i.e., rigid component of restraint and disinhibition) contribute to a positive relationship between attitudes toward thinness and forbidden foods (*discordant* attitudes). Therefore, the discordance between attitudes observed in the overall sample (i.e., positive association between attitudes toward thinness and forbidden foods) seemed to characterize primarily participants with problematic eating behaviors (rigid restraint and disinhibition), given that women whose eating behaviors were not problematic (flexible restraint) were characterized by congruent attitudes.

To our knowledge, the congruence or discordance between implicitly assessed attitudes had not previously been explored. However, one study that also used a nonrelative measure to implicitly assess attitudes explored discrepancies between explicitly and implicitly assessed attitudes in the field of dietary restraint and observed that restrained eaters, and not unrestrained eaters, exhibited discordant attitudes toward high-calorie foods (negative explicitly assessed attitudes, positive implicitly assessed attitudes) [22]. In line with our results, these findings suggest that a conflict between attitudes might influence the development and/or maintenance of problematic eating behaviors. It is indeed likely that discordant attitudes toward relevant goal-related constructs (e.g., a positive implicit attitude toward high-calorie foods in someone who is dieting to become thinner) will promote aversive emotional states and, ultimately, disordered eating (e.g., overeating, rigid restraint). In contrast, when attitudes are congruent, there is less internal tension, which allows for more flexible food control and intake (e.g., forbidden foods can be eaten in small quantities). Nevertheless, because our study is correlational in nature, the causal role of discordant implicitly assessed attitudes in the onset and maintenance of problematic eating remains tentative and should be addressed in future longitudinal studies.

It has been suggested that attitudes toward forbidden foods need to be negative in order to be congruent with positive attitudes toward thinness and the goal of losing weight [6]. However, the opposite relationship (i.e., discordance between attitudes) was observed in our sample. Therefore, it is possible that congruent attitudes exist only at the explicit level, at least among restrained eaters, who will disparage forbidden foods to promote their goal of dieting [6, 22]. Future studies should address this hypothesis.

Finally, although the investigation of interindividual differences make a substantial contribution by providing some explanations of the unpredicted results obtained for the overall sample, we cannot reach a conclusion regarding the nonsignificant results we obtained. In this sample, dysfunctional components of eating were related only to the link between attitudes toward thinness and forbidden foods (causing us to hypothesize that discordant attitudes existed), while the functional component was related only to the association with permitted foods (i.e., hypothesized congruent attitudes). However, the nonsignificant effects obtained for the other regression models do not necessarily mean that such effects were not present. Replication studies are necessary in order to draw more firmly based conclusions.

### Limitations

Some limitations on this study warrant further discussion. First, while this study focused on implicitly assessed attitudes, explicit measures of attitudes would also have been valuable. The consideration of explicitly assessed attitudes could lead to a better understanding of interindividual differences, given that, in certain cases, implicit and explicit measures of attitudes provide different information [8–10]. Moreover, explicit measures of attitudes would have allowed us to explore the discordance between explicitly and implicitly assessed attitudes toward similar stimuli (e.g., positive implicitly assessed attitudes and negative explicitly assessed attitudes toward forbidden foods), as well as the discordance between explicitly assessed attitudes and their links to symptoms of disordered eating.

Second, we used pictorial control stimuli in the two AMP versions to avoid a habituation effect due to the repetition of the same neutral stimulus (e.g., a gray square like the one used by Payne et al. [32]) and to increase the ecological validity of the tasks. These control stimuli had no connection with the concepts of interest for the AMP versions (respectively, body shape and food) and should therefore be irrelevant to the participants. Although they allowed us to take interindividual differences in responses into account (e.g., a general tendency to respond “pleasant”), we cannot exclude the possibility that relevant (thin and overweight bodies, high- and low-calorie foods) and irrelevant (shrubs, objects) stimuli were processed differently. Hence, the use of control stimuli related to the concept of interest might improve the methodology. For example, in the field of body image, in addition to positive (thin bodies) and negative (overweight bodies) “emotional” stimuli, the control stimuli could be “nonemotional” body-related stimuli, namely bodies evaluated as neither “thin” nor “overweight”, neither “positive” nor “negative”, but with a median judgment between the two poles (i.e., “zero-value” judgments).

Third, a general bias to respond “pleasant” might exist in AMP performances. Nevertheless, interindividual differences relativized this: some people might respond positively to a range of situations, whereas others might present the opposite tendency (to respond negatively) for the same situations [38]. Moreover, our findings showed that, as expected, responses in *control* trials did not significantly differ from chance level, highlighting that if such a bias exists, it was not present in all AMP conditions. Finally, interindividual differences in responses were taken into account in the statistical analyses to reduce the impact of such a bias.

Fourth, we did not use the classic TFEQ restraint scale, given that we wanted to explore the processes that underlie restraint (rigid and flexible cognitive controls). However, this choice makes our findings difficult to compare with previous studies that used the classic scale. Moreover, even if the internal reliability of the subscales was acceptable, it was lower than that of the classic scale, given the smallest number of items in the subscales (which have negatively influenced internal consistency) [39].

Finally, participants’ BMIs were based on self-reported measures. Estimation biases could therefore have influenced the measures. Nonetheless, previous research demonstrated that estimation biases are more likely to appear among people with “extreme” BMI [40, 41]. Given that such BMIs are rare in the current sample (nine participants have a BMI < 17.00 kg/m^2^ or > 30.00 kg/m^2^), we assume that estimation biases are minimal. Moreover, as BMI did not significantly correlate with the main AMP variables (i.e., AMP-assessed attitude toward body shape, permitted foods, or forbidden foods), it was not entered in the statistical analyses. Thus, our findings could not have been affected by such potential biases.

## Conclusions

Our results highlighted the importance of taking into account the congruence or discordance in patterns of attitudes toward body shape and food, as well as interindividual differences, in understanding problematic and nonproblematic eating behaviors. Focusing on interindividual differences seems essential to investigate the existence of different psychological patterns in a sample and highlight the mechanisms that may underlie a given subgroup’s behaviors. Further studies are nevertheless required to pursue the investigation of interrelationships between implicitly assessed attitudes toward body shape and food and obtain a more detailed picture of their influences on the onset and maintenance of eating behaviors.

These results have some critical clinical implications. Nowadays, in the field of eating disorders, schemas and implicit cognitions have been proposed to be a key focus for interventions [12, 30]. But our findings suggest a further step. First and foremost, given that discordance between implicitly assessed attitudes was found to be associated with problematic eating behaviors, the assessment of implicit representations toward body shape and food and their characterization in terms of congruent or discordant patterns seems necessary. Afterwards, interventions could target discordant implicit cognitions in order to treat problematic eating behaviors. For instance, Martijn et al. [42] proposed modifying positive implicit associations toward thinness (e.g., “thin – good”) through a conditioning procedure in which pictures of thin-ideal models were followed by a negative adjective (e.g., “fake”), emphasizing that models’ images are often retouched. By making thinness less positive, this intervention could reduce the internal attitudinal conflict among women who present positive implicitly assessed attitudes toward thinness and forbidden foods and perhaps allow them to engage in more flexible food control and intake, decreasing the frequency of problematic eating behaviors.

For definitions of specific terms, see Additional file 2.

## Additional files

Additional file 1:
**Figures representing associations between attitudes toward body shape and food at different levels of eating behaviors.** Three figures showing the regression lines illustrating the relations between AMP-assessed attitude toward body shape and (1) AMP-assessed attitude toward permitted foods at high, average, and low levels of the flexible cognitive control dimension of restraint; (2) AMP-assessed attitude toward forbidden foods at high, average, and low levels of the rigid cognitive control dimension of restraint; and (3) AMP-assessed attitude toward forbidden foods at high, average, and low levels of disinhibition. (PDF 474 kb) Additional file 2:
**Definitions of specific terms.** Table in which all definitions of specific terms are presented in order to facilitate the reader’s understanding. (PDF 178 kb)

## Abbreviations

AMP: Affect Misattribution Procedure
ANOVAs: analyses of variance
BMI: body mass index
IAT: Implicit Association Task
TFEQ: Three-Factor Eating Questionnaire

## Footnotes

1 In the present sample, no participant spoke Chinese or was familiar with this language, thereby fulfilling the inclusion criteria for the AMP.

2 The Mann–Whitney *U*-test was performed because the assumption of normality was not met.

3 Since this study focuses on restraint and disinhibition, the third dimension evaluated by the TFEQ (susceptibility to hunger, namely the perception of hunger and its impact on eating behaviors) was not used in the study.

4 The two manifestations of restraint and disinhibition did not significantly correlate with the predictor (AMP-assessed attitude toward body shape), respecting the criteria recommended by Kraemer et al. [43] for moderating variables. These variables were therefore considered as moderators when the interaction with the predictor had a significant effect on the outcome.

5 As BMI and age did not significantly correlate with the three main variables in the regression models (i.e., AMP-assessed attitude toward body shape, AMP-assessed attitude toward permitted foods and AMP-assessed attitude toward forbidden foods), they were not entered as control variables in the regression models to avoid reducing the statistical power.

6 As the Mauchly’s sphericity test (which evaluates whether the sphericity assumption associated with the repeated measures ANOVAs is met) was significant, the *F*-test was adjusted by the Huynh-Feldt correction, as needed.

7 Variables entered in the regression models were centered and the interaction term was computed with the centered variables. To test whether the main effect of the AMP-assessed attitude toward body shape was significant at a low level of flexible control, we reran the same regression model, except that the centered moderating variable was replaced by “centered flexible control +1 *SD*”. To explore whether the main effect was significant at a high level of flexible control, we replaced the centered moderating variable by “centered flexible control –1 *SD*”.

8 *b** corresponds to the standardized regression coefficient. *SE* corresponds to the standard error of *b**.
