# Supplementary material for: Implicitly assessed attitudes toward body shape and food: the moderating roles of dietary restraint and disinhibition
Source: J Eat Disord. 2015 Dec 8;3:47. doi: 10.1186/s40337-015-0085-8 (PMC4672544; doi:10.1186/s40337-015-0085-8)
Supplement: Additional file 1: — Figures representing associations between attitudes toward body shape and food at different levels of eating behaviors. Three figures showing the regression lines illustrating the relations between AMP-assessed attitude toward body shape and (1) AMP-assessed attitude toward permitted foods at high, average, and low levels of the flexible cognitive control dimension of restraint; (2) AMP-assessed attitude toward forbidden foods at high, average, and low levels of the rigid cognitive control dimension of restraint; and (3) AMP-assessed attitude toward forbidden foods at high, average, and low levels of disinhibition. (PDF 474 kb) [file 40337_2015_85_MOESM1_ESM.pdf]

Additional file 1

Figures representing associations between attitudes toward body shape and food at different levels of eating behaviors

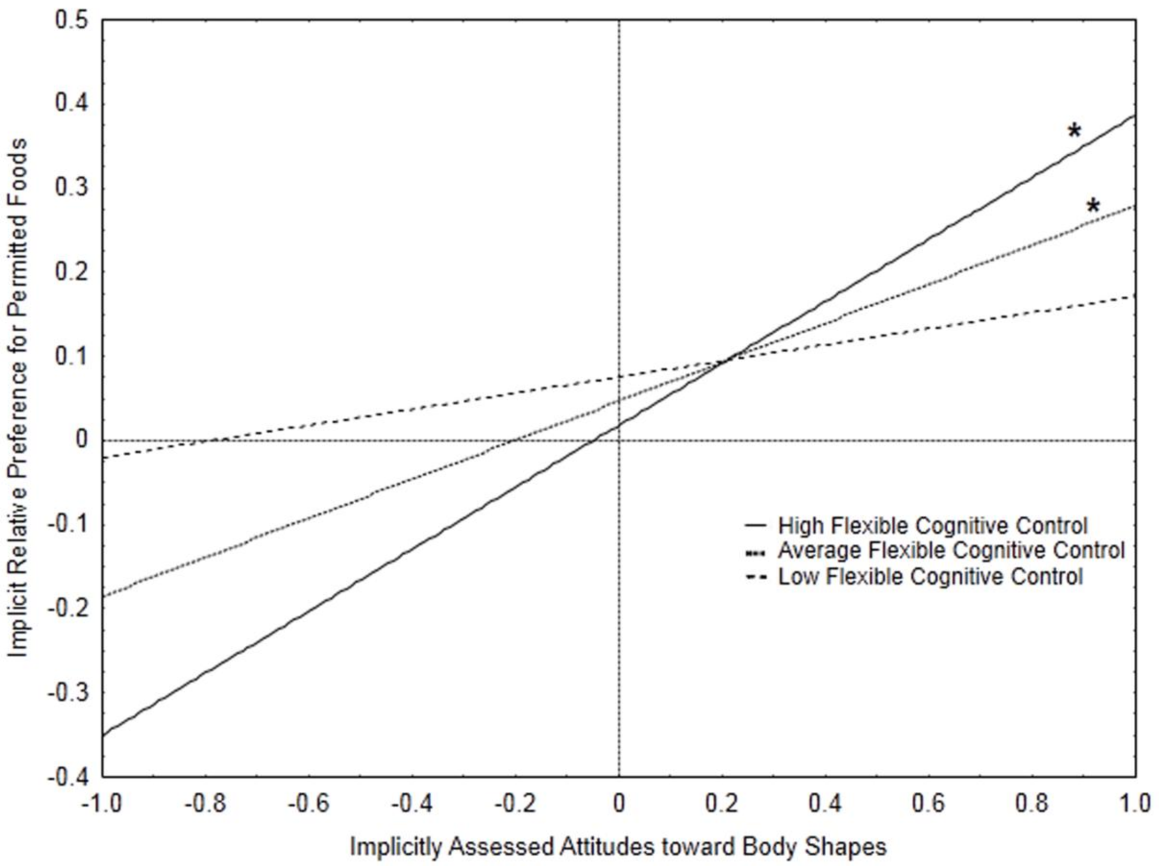

Figure 1. Association between attitudes toward body shape and food at different levels of flexible control.

The figure shows the regression lines illustrating the relations between AMP-assessed attitude toward body shape and AMP-assessed attitude toward permitted foods at high (+1 *SD*), average, and low (–1 *SD*) levels of the flexible cognitive control dimension of restraint. The scaling of both axes ranged from –1.00 to 1.00. For AMP-assessed attitude toward body shape, a positive score indicates a relative preference for thin bodies and a negative score indicates a relative preference for overweight bodies. For AMP-assessed attitude toward permitted foods, a positive score indicates a preference for permitted foods and a negative score indicates a preference for control images. Slopes were significant at average and high levels of flexible control. * $p < .01$.

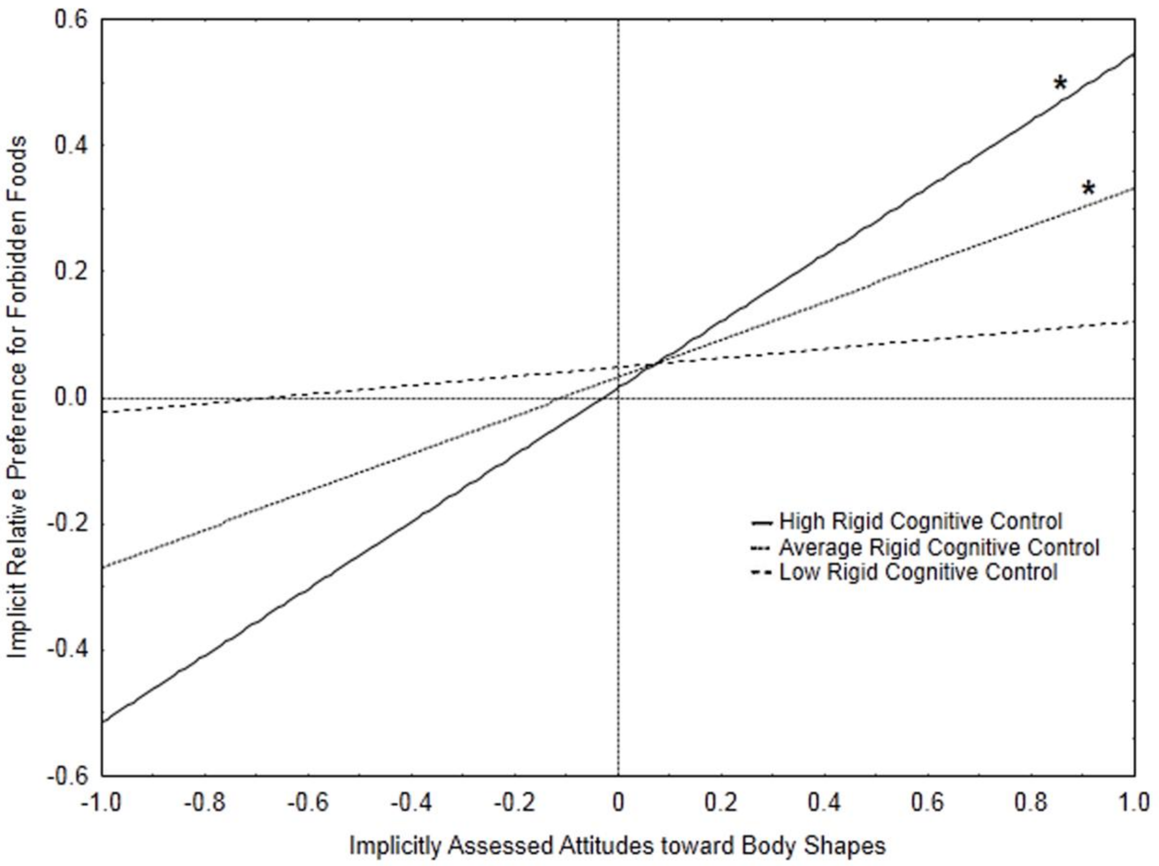

Figure 2. Association between attitudes toward body shape and food at different levels of rigid control.

The figure shows the regression lines illustrating the relations between AMP-assessed attitude toward body shape and AMP-assessed attitude toward forbidden foods at high (+1 *SD*), average, and low (–1 *SD*) levels of the rigid cognitive control dimension of restraint. The scaling of both axes ranged from –1.00 to 1.00. For AMP-assessed attitude toward body shape, a positive score indicates a relative preference for thin bodies and a negative score indicates a relative preference for overweight bodies. For AMP-assessed attitude toward forbidden foods, a positive score indicates a preference for forbidden foods and a negative score indicates a preference for control images. Slopes were significant at average and high levels of rigid control. * $p < .001$.

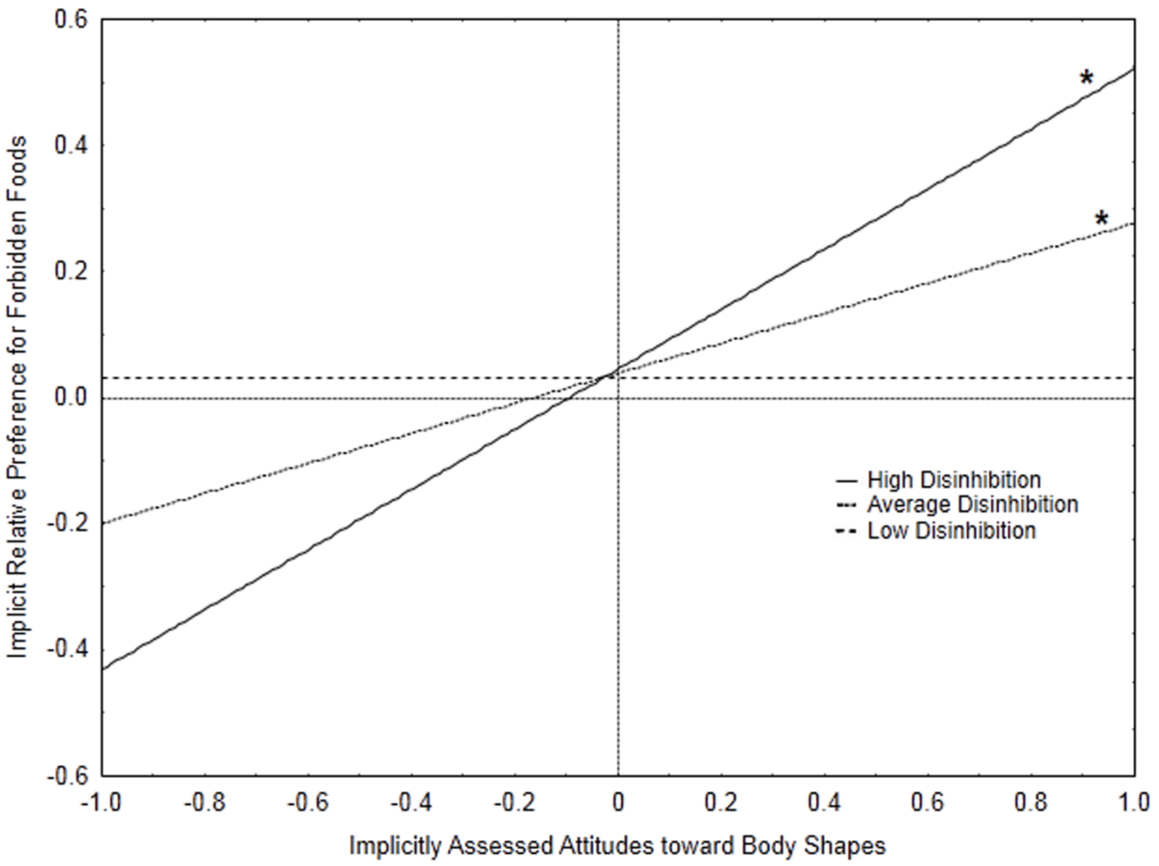

Figure 3. Association between attitudes toward body shape and food at different levels of disinhibition.

The figure shows the regression lines illustrating the relations between AMP-assessed attitude toward body shape and AMP-assessed attitude toward forbidden foods at high (+1 *SD*), average, and low (–1 *SD*) levels of disinhibition. The scaling of both axes ranged from –1.00 to 1.00. For AMP-assessed attitude toward body shape, a positive score indicates a relative preference for thin bodies and a negative score indicates a relative preference for overweight bodies. For AMP-assessed attitude toward forbidden foods, a positive score indicates a preference for forbidden foods and a negative score indicates a preference for control images. Slopes were significant at average and high levels of disinhibition. * $p < .01$.
