# Supplementary material for: Implicitly assessed attitudes toward body shape and food: the moderating roles of dietary restraint and disinhibition
Source: J Eat Disord. 2015 Dec 8;3:47. doi: 10.1186/s40337-015-0085-8 (PMC4672544; doi:10.1186/s40337-015-0085-8)
Supplement: Additional file 2: — Definitions of specific terms. Table in which all definitions of specific terms are presented in order to facilitate the reader’s understanding. (PDF 178 kb) [file 40337_2015_85_MOESM2_ESM.pdf]

Additional file 2

Definitions of specific terms

Term	Definition
**Interindividual differences**	Diversity of individuals on one dimension, trait, behavior, or characteristic (e.g., individuals who are high versus low on disinhibition).
	Attitudes
**Attitudes**	Representations stored in memory about the valence of a specific stimulus [1].
**Explicitly versus implicitly assessed attitudes**	**Explicitly assessed attitudes:** Attitudes assessed with measures (e.g., self-reports) that tap into reflective processes activated with voluntary cognitive control, meaning that they depend on responses and behaviors that participants are aware of, willing to report, and able to modify [1,7]. **Implicitly assessed attitudes:** Attitudes assessed with measures that reflect automatic processes activated without awareness or the possibility of controlling or modifying them [1,7].
	Conditions of the Affect Misattribution Procedure (AMP)
**Emotional versus control trials of the AMP**	**Emotional trials** – Thin trials: pictures of thin women; overweight trials: pictures of overweight women; permitted trials: pictures of “low-calorie” foods; forbidden trials: pictures of “high-calorie” foods. **Control trials –** primes/pictures (shrubs, everyday objects) have no connection with the concepts of interest for the AMP versions (body shape and food); “baseline trials” that allow interindividual differences in responses to be taken into account (e.g., tendency to respond in a similar way, such as showing “pleasant responses” through conditions) [32].

**Terms**	**Definitions**
	Interpretations of the AMP outcomes
**Positive versus negative AMP-assessed attitude**	**Positive attitude:** Inferred from an AMP condition in which participants were most likely to evaluate the Chinese characters as pleasant. In the present study, this was inferred from a condition in which judgments were significantly more pleasant in comparison to the control condition and the chance level of .50. Might be interpreted in terms of liking ("I like permitted food") [10]. **Negative attitude:** Inferred from an AMP condition in which participants were least likely to evaluate the Chinese characters as pleasant. In the present study, this was inferred from a condition in which judgments were significantly less pleasant in comparison to the control condition and the chance level of .50. Might be interpreted in terms of disliking ("I dislike overweight bodies/I like less overweight than control images").
**Congruent versus discordant attitudes**	**Congruent attitudes:** Refers to compatible attitudes. **Discordant attitudes:** Refers to incompatible attitudes or attitudes characterized by a conflict. Both could be inferred from the coherence between (1) attitudes toward the same concept but at different levels (i.e., explicitly versus implicitly assessed; examples: *congruent attitudes* – positive explicitly and implicitly assessed attitudes toward permitted foods; *discordant attitudes* – negative explicitly assessed and positive implicitly assessed attitudes toward forbidden foods); and (2) attitudes toward different concepts but at the same level (examples: *congruent attitudes* – positive explicitly assessed attitudes toward thinness and permitted foods; *discordant attitudes* – positive implicitly assessed attitudes toward thinness and forbidden foods).
	Dimensions of the Three-Factor Eating Questionnaire (TFEQ)
**Flexible cognitive control**	Functional component of restraint; nuanced attitude toward eating, in which "forbidden" foods can be eaten in small quantities [29].
**Rigid cognitive control**	Dysfunctional component of restraint; a dichotomous, all-or-nothing attitude toward eating [29].
**Disinhibition**	Dysfunctional component of eating; tendency to lose control over eating behaviors while experiencing adverse emotional states [29].
